# Overexpression of biotin synthase and biotin ligase is required for efficient generation of sulfur-35 labeled biotin in *E. coli*

**DOI:** 10.1186/1472-6750-10-73

**Published:** 2010-10-11

**Authors:** Teegan A Delli-Bovi, Maroya D Spalding, Sean T Prigge

**Affiliations:** 1 Department of Biochemistry and Molecular Biology, Johns Hopkins Bloomberg School of Public Health, Baltimore, MD 21205 USA; 2 Department of Molecular Microbiology and Immunology, Johns Hopkins Bloomberg School of Public Health, Baltimore, MD 21205 USA

## Abstract

**Background:**

Biotin is an essential enzyme cofactor that acts as a CO_2 _carrier in carboxylation and decarboxylation reactions. The *E. coli *genome encodes a biosynthetic pathway that produces biotin from pimeloyl-CoA in four enzymatic steps. The final step, insertion of sulfur into desthiobiotin to form biotin, is catalyzed by the biotin synthase, BioB. A dedicated biotin ligase (BirA) catalyzes the covalent attachment of biotin to biotin-dependent enzymes. Isotopic labeling has been a valuable tool for probing the details of the biosynthetic process and assaying the activity of biotin-dependent enzymes, however there is currently no established method for ^35^S labeling of biotin.

**Results:**

In this study, we produced [^35^S]-biotin from Na^35^SO_4 _and desthiobiotin with a specific activity of 30.7 Ci/mmol, two orders of magnitude higher than previously published methods. The biotinylation domain (*Pf*BCCP-79) from the *Plasmodium falciparum *acetyl-CoA carboxylase (ACC) was expressed in *E. coli *as a biotinylation substrate. We found that overexpression of the *E. coli *biotin synthase, BioB, and biotin ligase, BirA, increased *Pf*BCCP-79 biotinylation 160-fold over basal levels. Biotinylated *Pf*BCCP-79 was purified by affinity chromatography, and free biotin was liberated using acid hydrolysis. We verified that we had produced radiolabeled biologically active [*D*]-biotin that specifically labels biotinylated proteins through reuptake in *E. coli*.

**Conclusions:**

The strategy described in our report provides a simple and effective method for the production of [^35^S]-biotin in *E. coli *based on affinity chromatography.

## Background

Biotin, or vitamin H, was first identified as a yeast growth factor over 100 years ago[1] and was subsequently isolated from egg yolk[2] and liver[3]. It is an essential cofactor for a small family of enzymes that catalyze carboxylation and decarboxylation reactions, in which biotin serves as a covalent attachment site for CO_2_[4]. The number of biotinylated proteins varies from one to five in different organisms[5]. Biotin-dependent enzymes include acetyl-CoA carboxylase, pyruvate carboxylase, propionyl-CoA carboxylase, methylcrotonyl-CoA carboxylase, geranoyl-CoA carboxylase, oxaloacetate decarboxylase, methylmalonyl-CoA decarboxylase, transcarboxylase and urea amidolyase[6]. These enzymes participate in central metabolic processes such as gluconeogenesis, lipogenesis, amino acid metabolism and energy transduction. The most widespread biotin-dependent enzyme is acetyl-CoA carboxylase (ACC)[7], which catalyzes the ATP-dependent transfer of a carboxyl group from carbonate to acetyl-CoA to form malonyl-CoA, in the first committed step of fatty acid biosynthesis[8].

ACC is the only biotinylated enzyme in *Escherichia coli*[9], and it exists as a complex of four proteins: biotin carboxylase (BC), carboxyl transferase alpha and beta chains (CT), and biotin carboxy carrier protein (BCCP)[10]. The BC subunit is responsible for transferring a carboxyl group from a substrate (carbonate) to the biotin prosthetic group, which is covalently attached to a conserved lysine in the BCCP subunit[11]. The CT serves to transfer the carboxyl group from biotin to acetyl-CoA, forming malonyl-CoA[12]. Biotin is attached to BCCP by a dedicated biotin protein ligase, BirA. This enzyme catalyzes the ATP-dependent formation of an amide linkage between the carboxyl group of biotin and the ε-amino group of a specific lysine residue in BCCP[4]. The primary structure of biotinylation domains exhibits a high degree of similarity across species. The biotinylated lysine residue occurs in a conserved AMKM tetrapeptide, and a minimum of 75-80 residues surrounding this motif are required for recognition by BirA. Biotin protein ligases show significant cross reactivity between species, i.e. bacterial ligases biotinylate mammalian apo-proteins, and vice versa[4].

*E. coli *can scavenge biotin from the environment, or synthesize it *de novo *from a pimeloyl-CoA precursor. The enzymes of the biosynthetic pathway are encoded by the *bio *operon (*bioABCFD*)[13]. The final step, insertion of sulfur into desthiobiotin to form biotin, is catalyzed by biotin synthase (BioB). This enzyme contains two iron-sulfur clusters, a [4Fe-4S] cluster common to all radical SAM (*S*-adenosyl-L-methionine) enzymes which facilitates the reductive cleavage of SAM[14], and a [2Fe-2S] cluster which donates the sulfur atom to desthiobiotin[15].

The metabolic origins of biotin and its attachment to proteins have been tracked using carbon, sulfur and hydrogen isotopes[16-18]. Currently [^3^H]-biotin is commercially available, but is not feasible to use as a metabolic label in some systems due to the low sensitivity of ^3^H. Previous attempts have been made to label biotin with ^35^S using intact cells of *Saccharomyces cerevisiae*[19], *Aspergillus niger*[20] and *Rhodotorula glutinis*[21], however these attempts were hampered by poor yield, low specific activity of biotin, or the predominance of oxidized biotin species.

**Figure 1:**
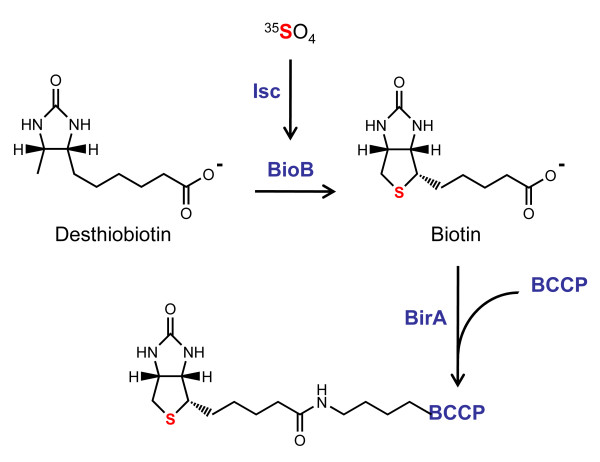
**Synthesis of radiolabeled biotin in *E. coli***. The *E. coli *biotin synthase (BioB) catalyzes the insertion of sulfur into desthiobiotin to form biotin, which is then ligated to the biotin carboxy carrier protein (BCCP) by the biotin protein ligase (BirA). The sulfur is donated from a [2Fe-2S] cluster on BioB which is formed by iron sulfur cluster biogenesis (Isc) proteins. *E. coli *supplemented with desthiobiotin and ^35^SO_4 _will produce small quantities of radiolabeled biotin bound to BCCP. The *P. falciparum *BCCP, which does not contain the amino acid cysteine, was overexpressed in the methionine auxotroph *E. coli *strain B834(DE3) to prevent the incorporation of ^35^S into the BCCP protein. BirA, BioB and the Isc proteins were overexpressed to increase the yield of radiolabeled biotin.

We developed a strategy to synthesize [^35^S]-biotin from desthiobiotin and Na^35^SO_4 _in *E. coli *(Figure 1). We expressed a 79-residue biotinylation domain (*Pf*BCCP-79) from the *Plasmodium falciparum *ACC protein, and purified *Pf*BCCP-79 by affinity chromatography. We found that coexpression of the *E. coli *biotin synthase, BioB, increased *Pf*BCCP-79 biotinylation over basal levels, however, coexpression of both BioB and the *E. coli *biotin ligase, BirA, was required for efficient biotinylation of *Pf*BCCP-79. In these experiments, the BioB expression plasmid could not be substituted with a plasmid expressing the *Azotobacter vinelandii *Isc (Iron Sulfur Cluster) proteins, suggesting that remetalation of endogenous BioB did not significantly increase biotin synthase activity. Biotin was liberated from pure *Pf*BCCP-79 using acid hydrolysis, and was quantified in a bioassay based on the growth of a biotin auxotroph *E. coli *strain. We measured reuptake of biotin in *E. coli *to verify that we had produced biologically active, radiolabeled [*D*]-biotin, and found that we were able to specifically label the *E. coli *BCCP. From a 10 ml culture of cells supplemented with 1 mCi Na^35^SO_4 _we obtained 37 pmol of [^35^S]-biotin, with a specific activity of 30.7 Ci/mmol.

## Results and Discussion

### Expression of a biotin-domain in *E. coli*

Biotin biosynthesis in *E. coli *is a tightly regulated process, and results in about 200 molecules of protein-bound biotin per cell[22]. To increase the yield of biotin production in *E. coli*, we expressed a biotin carrier protein, which contained an affinity tag to aid in its purification. Both *E. coli *and the malaria parasite *Plasmodium falciparum *encode a single biotinylated protein in their genome, the acetyl-CoA carboxylase (ACC). In *E. coli*, the enzyme exists as a complex of four proteins, while in *P. falciparum*, the ACC is a single large polypeptide. Despite the difference in structure, the domains of the *P. falciparum *ACC are homologous to the protein subunits of the *E. coli *ACC complex. Biotin is conjugated to a particular lysine in the biotin carboxy carrier protein (BCCP) domain. We selected the *P. falciparum *BCCP as a biotinylation substrate because it does not contain cysteine residues, which would incorporate the ^35^S label and complicate the purification and quantification of radiolabeled biotin. The *E. coli *BCCP contains a single cysteine at amino acid 116, which is only four amino acids away from the conserved AMKM biotinylation motif. Therefore, mutation of this residue may interfere with the folding of the protein or the biotinylation reaction. The ~80-residue biotinylation sites of the BCCP orthologs share 48% sequence similarity, so we expected that the *E. coli *biotin ligase would recognize and biotinylate the *P. falciparum *protein *in vivo*.

**Figure 2:**
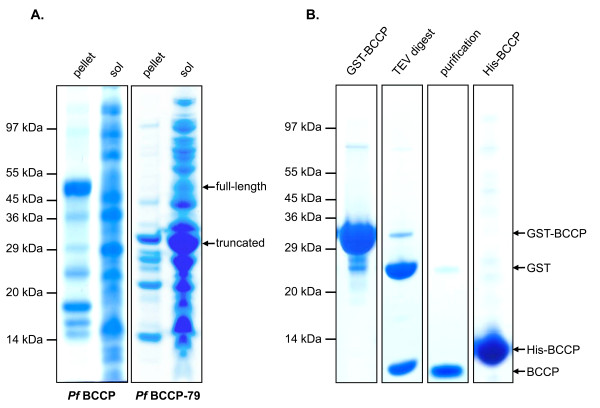
**Expression and solubility of *Pf*BCCP**. (A) Coomassie-stained SDS-PAGE gel showing soluble and insoluble fractions from cells expressing full length or truncated *Pf*BCCP. The full-length 214-residue biotin carboxy carrier protein (BCCP) domain of the *P. falciparum *ACC gene (*Pf*BCCP) and a 79-residue fragment containing the biotinylation site (*Pf*BCCP-79) were expressed as GST fusion proteins in BL21-Star(DE3) cells. Whole cell lysate was separated into insoluble (pellet) and soluble (sol) fractions by centrifugation. The truncated fragment was predominantly soluble. (B) SDS-PAGE gel showing GST-tagged *Pf*BCCP-79 (GST-BCCP) purified by affinity chromatography on a GSTrap™ Fast-Flow column (lane 1) and cleaved *in vitro *by tobacco etch virus (TEV) protease (lane 2). The sample was reapplied to the column to remove free GST and the purified protein (lane 3) was used for production of antisera in rabbits. *Pf*BCCP-79 was also purified with a six-histidine tag (His-BCCP) on a metal chelating column charged with NiCl_2 _(lane 4).

Initially, the entire 214-residue BCCP domain (*Pf*BCCP) of the *P. falciparum *ACC was cloned into a modified pGEX-4T-3 vector (pGEXT), which encodes a GST fusion protein that can be cleaved by tobacco etch virus (TEV) protease. Expression tests in *E. coli *indicated that the full-length *Pf*BCCP domain was insoluble (Figure 2A, lane 1). We then cloned only the C-terminal 79 residues of BCCP (*Pf*BCCP-79) into pGEXT, and when we expressed this fragment in *E. coli *we found that it was soluble (Figure 2A, lane 4). These residues correspond to the 80-residue biotinylation domain found in the crystal structure of *E. coli *BCCP [PDB:1BDO]. Chapman-Smith and coworkers demonstrated that a similar fragment, the C-terminal 87 residues of *E. coli *BCCP, acts as a stable domain that is biotinylated *in vivo *by the biotin ligase, BirA[23].

*Pf*BCCP-79 was expressed as a GST fusion protein in BL21-Star(DE3) cells, and purified by affinity chromatography on a glutathione-Sepharose column, yielding 110 mg of protein from 1 L of culture (Figure 2B, lane 1). The fusion protein was cleaved *in vitro *by TEV protease (lane 2) and the sample was reapplied to the column to remove free GST. Our final sample did not contain any contaminants visible by Coomassie blue staining, except for a faint band corresponding to free GST (lane 3). Purified *Pf*BCCP-79 was concentrated to 1.2 g/L, and used for the production of specific antisera in rabbits.

Because the GST fusion protein is not ideal for labeling with ^35^S, due to the presence of multiple cysteines that would incorporate the radiolabel, we also expressed *Pf*BCCP-79 with an N-terminal six-histidine tag from plasmid pTDe010. pTDe010 encodes His_6_-tagged *Pf*BCCP-79 under control of a T7 promoter, with a kanamycin resistance cassette and a pMB1 origin of replication. His_6_-tagged *Pf*BCCP-79 was expressed in BL21-Star(DE3) cells, and purified on a metal chelate column charged with NiCl_2_, yielding 90 mg of protein from 2 L of culture (Figure 2B, lane 4). These data demonstrate that *Pf*BCCP-79 is soluble, expressed at high levels, and can be purified with high yield by affinity chromatography.

### Biotinylation of *Pf*BCCP-79

In order to evaluate whether *Pf*BCCP-79 is a valid biotin carrier for the purification of [^35^S]-biotin from *E. coli*, we tested whether it is biotinylated efficiently by the *E. coli *biotin ligase, BirA. It has been shown that *Ec*BCCP is not efficiently biotinylated when it is overexpressed, unless it is coexpressed with BirA[23]. In order to assess whether this was the case for *Pf*BCCP-79, we measured biotinylation levels of *Pf*BCCP-79 in cells that overexpress BirA from plasmid pCY216[23], which encodes the *E. coli birA *gene under control of an arabinose-inducible promoter in a chloramphenicol resistant plasmid. We considered expressing a cognate biotin ligase from *P. falciparum*, however, malaria parasites appear to encode two BirA paralogs (PF10_0409 and PF14_0573) and it is not clear which enzyme would be the appropriate ligase for *Pf*BCCP-79. We therefore chose to rely on the fact that there is a high level of cross-reactivity between biotin ligases and biotinylated proteins from divergent species[4].

**Figure 3:**
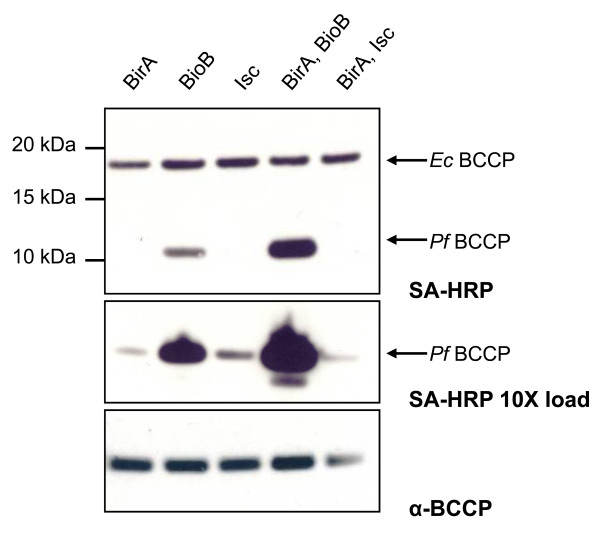
**Biotinylation of *Pf*BCCP-79**. Western blot analysis of B834(DE3) *E. coli *cell lysate from cultures expressing his_6_-tagged *Pf*BCCP-79, in combination with either the *E. coli *biotin ligase, BirA, the *E. coli *biotin synthase, BioB, or the iron sulfur cluster (Isc) proteins from *Azotobacter vinelandii*. Biotinylation levels of the *E. coli *BCCP (17kDa) and *P. falciparum *BCCP (11kDa) were assessed by affinity blotting with streptavidin-HRP (SA-HRP, upper panel). Biotinylation can be enhanced 160-fold by coexpressing *Pf*BCCP-79 with BirA and BioB. Loading samples ten times more concentrated shows that there was some biotinylation under all conditions (SA-HRP 10X load, middle panel). Re-probing the blot with *Pf*BCCP antiserum shows that *Pf*BCCP-79 was expressed at similar levels in all samples (α-BCCP, bottom panel).

All experiments were carried out using the methionine auxotroph *E. coli *strain B834(DE3). Although protein expression levels in this cell line were lower than in BL21-Star(DE3) cells, it was necessary to use a methionine auxotroph in order to prevent incorporation of ^35^S into methionine. The methionine residues cannot be mutated from BCCP proteins since the AMKM biotinylation site contains methionine amino acids. Cells were transformed with expression vectors pTDe010 and pCY216. Cultures of these cells were grown to log phase, protein expression was induced with IPTG and arabinose, and the cultures were supplemented with the biotin precursor desthiobiotin. Cells were harvested and biotinylation of *Pf*BCCP-79 was measured by affinity blotting using streptavidin-HRP (Figure 3, upper and middle panels). This probe also detected the endogenous *Ec*BCCP. As expected, levels of biotinylated *Ec*BCCP did not change between any of the conditions we tested, since *Ec*BCCP is fully biotinylated under normal conditions[24].

We found that overproduction of BirA did not appear to effect the efficiency of *Pf*BCCP-79 biotinylation (Figure 3, lane 1). This may be explained by the transcriptional regulation function of BirA. When BCCP is fully saturated with biotin the BirA-biotinyl-AMP intermediate accumulates, promoting the cooperative dimerization of BirA; this complex binds to the biotin operator and represses transcription of biotin biosynthetic genes[25,26]. In this way, biotin synthesis and protein biotinylation are tightly coupled, and there is little free intracellular biotin[27]. Overexpression of BirA may increase the amount of the repressor species, and thus shut down biotin biosynthesis. This hypothesis implies that the dynamics of biotinylation differ between the *E. coli *and *P. falciparum *substrates, that is, if the efficiency of *Pf*BCCP-79 biotinylation is significantly slower than that of *Ec*BCCP, the BirA-biotinyl-AMP repressor will accumulate even in the presence of excess apo-*Pf*BCCP-79. The fact that *Ec*BCCP biotinylation levels did not change between different conditions supports this model.

To circumvent this problem, we coexpressed *Pf*BCCP-79 with both BirA and the biotin synthase, BioB. BioB catalyzes the final step in the biotin biosynthetic pathway, the insertion of sulfur into desthiobiotin to form biotin[28]. We measured *Pf*BCCP-79 biotinylation in cells expressing BioB from plasmid pSPr059, which encodes the *E. coli bioB *gene under control of the λP_L/tetO _promoter in an ampicillin resistant plasmid. We found that coexpression of *Pf*BCCP-79 with BioB alone increased biotinylation 16-fold over the level seen with BirA alone (Figure 3, lane 2), while coexpression with both BioB and BirA resulted in an additional 10-fold increase in biotinylation (lane 4).

BioB donates sulfur from a [2Fe-2S] cluster to desthiobiotin in a suicide mechanism[29]. If regeneration of the iron-sulfur cluster is a limiting step in this pathway, then biotinylation efficiency can be increased by facilitating the remetalation of BioB. The Isc (Iron Sulfur Cluster) proteins from *Azotobacter vinelandii *comprise the iron-sulfur cluster biogenesis pathway in this organism[30] and have been shown to increase the yield of properly metalated biotin synthase when expressed in *E. coli*[31]. We tested the effect on biotinylation levels when we added plasmid pDB1282 to our cell line, which encodes the essential genes of the Isc operon from *A. vinelandii *(*iscS, iscU, iscA, hscB, hscA *and *fdx*)[30] under control of an arabinose-inducible promoter with an ampicillin resistance cassette. We found that overproduction of *Pf*BCCP-79 with the *Av*Isc proteins, either alone or in combination with BirA, did not have a significant effect on biotinylation levels (Figure 3, lanes 3 and 5). These results indicate that remetalation of endogenous BioB by the Isc proteins was less effective in promoting biotin synthesis than overexpression of BioB, and thus the formation of iron-sulfur clusters on BioB is not a limiting step in biotin synthesis.

An alternative explanation is that the *Av*Isc proteins are not expressed at sufficient levels to affect *Pf*BCCP-79 biotinylation. These experiments were carried out in minimal media containing glucose as the carbon source. Since glucose inhibits the uptake of pentose sugars such as arabinose[32], the induction of BirA and the *Av*Isc proteins could be affected by this medium. We conducted the experiments shown in Figure 3 substituting glucose with 0.4% glycerol and found that biotinylation in cells expressing BirA tripled, however, there was no effect in cells expressing the *Av*Isc proteins, and only a modest increase of 8% in cells expressing both BirA and BioB (data not shown). Thus, removing glucose from the medium does slightly increase biotinylation due to increased expression of BirA, but not *Av*Isc proteins. Although glucose does affect the expression of BirA by arabinose, the long low temperature induction used in our experiments largely compensates for this effect. Indeed, we observe protein bands by SDS-PAGE corresponding to BirA and certain Isc proteins which are only present after induction with arabinose (data not shown).

Loading samples ten times more concentrated showed that there was some biotinylated *Pf*BCCP-79 under all the conditions tested (Figure 3, middle panel). Western blot analysis using *Pf*BCCP antiserum showed that the protein is expressed at similar levels in all the cultures (Figure 3, lower panel). Together, these results indicate that *Pf*BCCP-79 can be expressed in *E. coli*, and is biotinylated by the *E. coli *biotin ligase. We can increase the yield of biotin production by overexpressing key enzymes of the biosynthetic pathway. We bypassed the negative feedback loop that regulates the biotin operon by overexpressing the biotin synthase, BioB, under a constitutive promoter, and supplementing the culture with the BioB substrate, desthiobiotin. We found that it was also necessary to overexpress the biotin ligase, BirA, for efficient biotinylation of *Pf*BCCP-79.

### Purification of *Pf*BCCP-79

**Figure 4:**
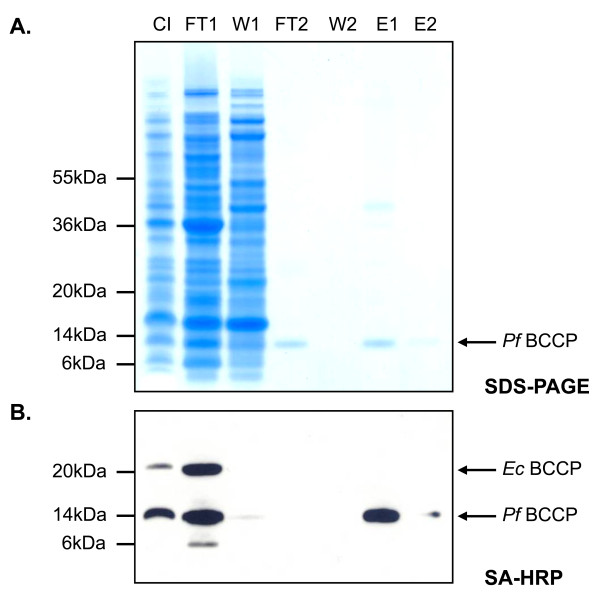
**Purification of *Pf*BCCP-79**. *Pf*BCCP-79 was coexpressed with BirA and BioB in B834(DE3) cells and purified by affinity chromatography. (A) Coomassie-stained SDS-PAGE gel showing fractions collected from each purification step. (B) Affinity blot using streptavidin-HRP to detect biotinylated proteins. Biotinylated *Pf*BCCP-79 was present in the flow through from the metal chelate column and in the elution. The gel samples are labeled as follows: column input (CI); flow through from metal chelate column (FT1); 30 mM imidazole wash (W1); flow through from monomeric avidin column (FT2); buffer wash (W2); elution in 50 mM glycine pH 2.9 (E1); elution in 50 mM glycine pH 1.9 (E2).

We purified His_6_-tagged *Pf*BCCP-79 from whole cell lysate using affinity chromatography. We expressed *Pf*BCCP-79 in B834(DE3) cells, together with BioB and BirA. Cells were grown to log phase at 37°C and protein expression was induced with IPTG and arabinose. Cultures were transferred to 20°C, and after one hour, the substrate desthiobiotin was added. They were maintained at 20°C for an additional 10 hours, after which cells were harvested and lysed. Cell lysate was cleared by centrifugation and applied to a metal chelate column charged with NiCl_2_. The column was washed with 30 mM imidazole, 1 M NaCl, and 1% Triton X-100 to remove proteins that interact weakly or non-specifically. Bound proteins were then eluted with 400 mM imidazole onto a column packed with monomeric avidin resin, and the column was washed with buffer. Bound proteins were eluted with 50 mM glycine pH 2.9, which denatures the avidin so that it releases biotin. It is typical to elute from an avidin column with high concentrations of biotin, but this is undesirable in our case since it would add large amounts of unlabeled biotin to the sample. The advantage of using avidin resin is that it separates apo-*Pf*BCCP-79 from holo-*Pf*BCCP-79, allowing us to estimate the proportion of the protein that is biotinylated. The unbiotinylated protein was collected in the flow through from the avidin affinity column (Figure 4A, lane 4), while the biotinylated protein was collected in the elution fractions (Figure 4A, lanes 6 and 7). Samples collected at each step in the purification were separated by SDS-PAGE and stained with Coomassie Blue dye, and densitometry analysis was carried out using ImageJ[33,34].

By comparing the flow through (FT2) and elution fractions (E1/E2) from the avidin column, we estimated that 40% of the *Pf*BCCP-79 from the metal chelate column was binding to the avidin column. This suggests that *in vivo *less than half of the protein is biotinylated, or alternatively, that biotinylated protein did not bind to the column for some reason. To address this question, we transferred the proteins to nitrocellulose membrane for affinity blotting using streptavidin-HRP. No biotinylated proteins were detected in FT2, confirming that the unbound *Pf*BCCP-79 was not biotinylated (Figure 4B). This experiment also revealed that approximately half (49%) of the biotinylated protein did not bind to the metal chelate column (Figure 4B, lane 2), perhaps due to slow kinetics of binding. To address this question, we reapplied the flow through to a clean metal chelate column, and observed that no additional *Pf*BCCP-79 bound the column (data not shown). Thus, this protein cannot bind to the column, probably due to degradation of the affinity tag. Indeed, the lower band near 6 kDa in FT1 is likely a degradation product of biotinylated *Pf*BCCP-79. One way to address this problem, and increase the yield from the purification, would be to tag the protein on both the N- and C-termini. We estimated that 18% of the *Pf*BCCP-79 present in the cell lysate was recovered in the elution. Overall, these data show that *Pf*BCCP-79 is a valid biotinylation substrate in *E. coli *and can be purified from cell lysate by affinity chromatography.

### Production of [^35^S]-biotin

**Figure 5:**
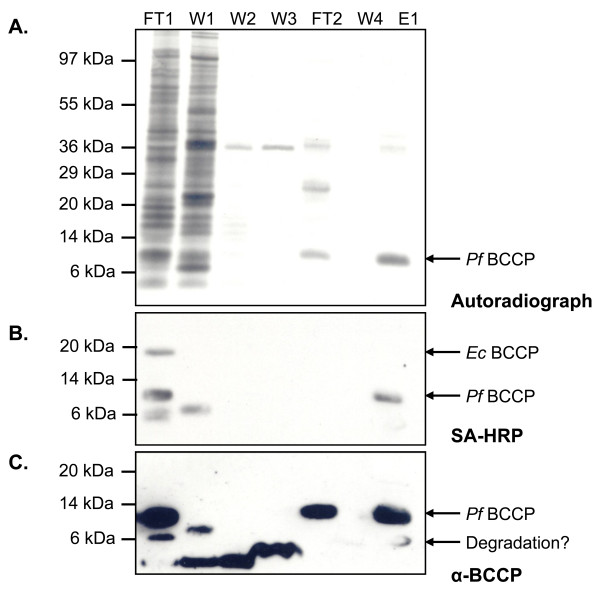
**Purification of [^35^S]-biotinylated *Pf*BCCP-79**. *Pf*BCCP-79 was coexpressed with BirA and BioB in B834(DE3) cells in the presence of Na^35^SO_4 _and desthiobiotin, and purified by affinity chromatography. (A) Autoradiograph of a SDS-PAGE gel showing samples collected at each purification step. (B) Affinity blot analysis with streptavidin-HRP to detect biotinylated proteins. (C) Western blot analysis with *Pf*BCCP antiserum. The gel samples are labeled as follows: flow through from metal chelate column (FT1); 30 mM imidazole wash (W1); 1 M NaCl wash (W2); 1% triton X-100 wash (W3); flow through from monomeric avidin column (FT2); buffer wash (W4); elution in 50 mM glycine pH 2.9 (E1).

After validating the conditions for purifying His_6_-tagged *Pf*BCCP-79 from *E. coli*, we used this procedure to generate [^35^S]-biotin. We grew 10 ml cultures of B834(DE3) cells to log phase at 37°C, then transferred the cultures to 20°C and induced protein expression with IPTG and arabinose. After one hour, 1 mM of the biotin precursor desthiobiotin and 1 mCi of Na^35^SO_4 _were added to the culture. The culture was maintained at 20°C for an additional 10 hours. Cells were then harvested and His_6_-tagged *Pf*BCCP-79 was purified from whole cell lysate as described above. Samples from each step of the purification were collected and analyzed by SDS-PAGE and autoradiography (Figure 5A). The 11.3 kDa band in the elution (E1) corresponds to [^35^S]-biotin-*Pf*BCCP-79. The only source of radioactivity in the protein should be from the biotin prosthetic group, because *Pf*BCCP-79 has no cysteines that could incorporate the radiolabel, and the B834(DE3) strain is a methionine auxotroph and can only utilize the unlabeled methionine added to the medium. Therefore, the band of similar size to *Pf*BCCP-79 in FT2 is either another protein of unknown identity, or biotinylated BCCP in which the biotin has been damaged in such a way that it is not recognized by avidin. Affinity blotting using streptavidin-HRP showed that there is no detectable biotinylated *Pf*BCCP-79 in FT2 (Figure 5B).

We also analyzed these samples using anti-*Pf*BCCP antiserum (Figure 5C). The antiserum detected the same ~6 kDa band in FT1 that we observed in the previous experiment. This indicates that the fragment is a degradation product of the *Pf*BCCP-79 fusion protein, and not derived from the endogenous *E. coli *BCCP. This band is also present in the purified sample (E1), suggesting that some degradation occurs during the purification process. We carried out densitometry analysis of this western blot to determine the proportion of *Pf*BCCP-79 present in each sample, and our results were consistent with those from the previous experiment. We estimated that 56% of the protein did not bind the MC column (versus 49% in the previous experiment), and was collected in FT1. An additional 6% was removed in the first wash with 30 mM imidazole. The remaining unbiotinylated *Pf*BCCP-79 was collected in the flow through from the avidin column, which amounted to 18% of total protein. Our purified sample contained 21% of the starting amount of *Pf*BCCP-79 in the cell lysate (compared to 18% in the previous experiment), corresponding to 9.9 μg of *Pf*BCCP-79. We also repeated our calculation of the biotinylation efficiency based on these results. We estimated that 54% of the total *Pf*BCCP-79 was biotinylated, which was higher than our previous estimate of 40%. This difference may be due to differences between the two experiments, or error associated with comparing results between SDS-PAGE and western blot images.

### Verification and quantification of biologically active [^35^S]-biotin in *E. coli*

**Figure 6:**
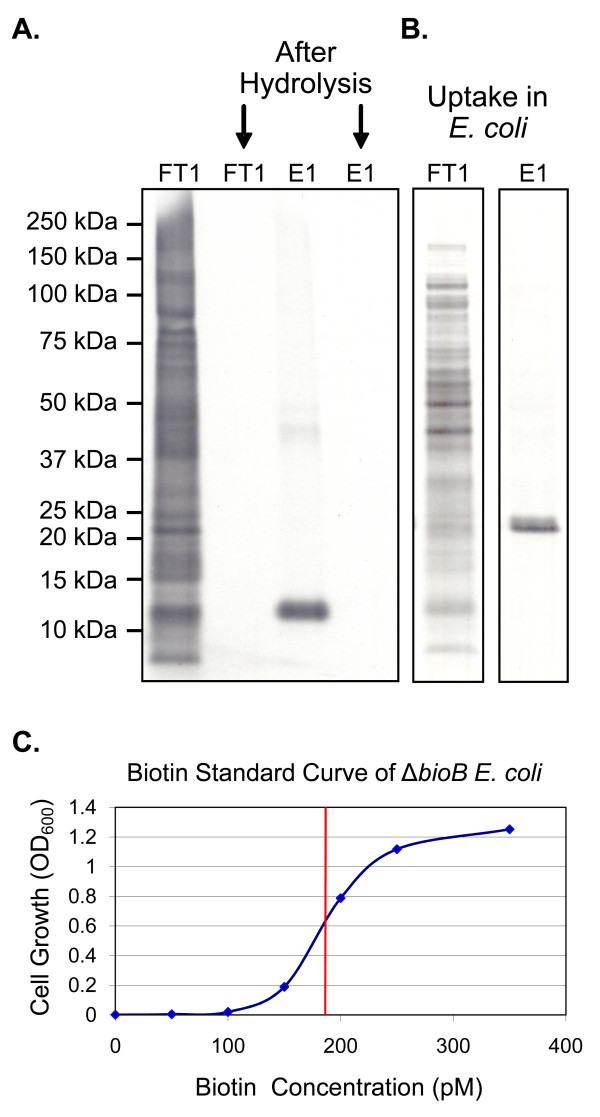
**Liberation of biologically active [^35^S]-biotin, and its verification and quantification in *E. coli***. (A) Autoradiograph of a SDS-PAGE gel showing acid hydrolysis of the flow through from the metal chelate column (FT1) and the elution fraction containing ^35^S-labeled *Pf*BCCP-79 (E1). (B) Autoradiograph of cell lysate from a Δ*bioB E. coli *strain cultured with the protein hydrolysates shown in panel A. The 17 kDa BCCP protein from *E. coli *is specifically labeled by the E1 hydrolysate, showing that [^35^S]-biotin is taken up and incorporated into cellular proteins. (C) Standard curve showing cell growth of Δ*bioB E. coli *cultures supplemented with increasing concentrations of biotin. This curve was used to quantify the concentration (indicated by the red line) of [^35^S]-*D*-biotin produced.

Acid hydrolysis was used to degrade proteins in the sample, thus liberating free biotin. We digested both FT1 and E1 in 5 M HCl for 8 hours at 95°C. FT1 served as the negative control, since this sample contained proteins that have incorporated ^35^S-labeled cysteine residues. We compared these samples before and after hydrolysis using SDS-PAGE and autoradiography. The disappearance of all bands in both samples after hydrolysis shows that proteins were completely digested under these conditions (Figure 6A). We then wished to verify that the [^35^S]-biotin we produced was biologically available to the cell. To this end, we used the biotin auxotroph *E. coli *strain Keio JW0758 in which the biotin synthase (*bioB*) gene had been replaced with a kanamycin resistance cassette[35,36], similar to the strain employed by Hwang and coworkers[37]. We supplemented cultures of Δ*bioB E. coli *with either the FT1 or E1 hydrolysate. Cells were cultured in minimal media with no other source of biotin. Both protein hydrolysates were able to support cell growth, due to the presence of biotinylated *Pf*BCCP-79 and *Ec*BCCP in FT1. The cells were harvested, and proteins were separated by SDS-PAGE and analyzed by autoradiography. In cultures supplemented with E1 hydrolysate, a single band appeared on the autoradiograph, which corresponded in size to the 17 kDa *E. coli *BCCP. In contrast, cultures supplemented with FT1 hydrolysate incorporated [^35^S]-cysteine into all newly synthesized proteins (Figure 6B). These results demonstrate that the radiolabeled biotin in the sample is biologically active and available to cellular enzymes, and specifically labels biotinylated proteins.

The hydrolysate containing [^35^S]-biotin may be used directly for biotin labeling experiments, as the only source of radioactivity in the sample is from biotin. However, in some cases it may be desirable to further purify biotin from the hydrolyzed protein and other material in the sample. Silica gel flash chromatography or high performance liquid chromatography (HPLC) would be most suitable for this purpose.

We developed an assay to quantify the amount of biotin in the sample, using the same Δ*bioB E. coli *strain. If this strain is supplemented with known concentrations of biotin, it will grow at a rate proportional to the biotin in the culture, over a small range of biotin concentrations (between 0 pM and 400 pM). Based on the biotin concentrations that limited growth, we estimated that about 250 molecules of biotin are required per *E. coli *cell, in close agreement with a previous estimation of 200 molecules per cell[22]. These cultures were used to construct a standard growth curve (Figure 6C). Cultures supplemented with serial dilutions of the hydrolysate were grown in parallel, and the optical density of these cultures was compared to the standard curve in order to determine the concentration of biotin in the original sample. We found that a 2 mL culture of Δ*bioB E. coli *supplemented with 5 μl of *Pf*BCCP-79 hydrolysate grew to an OD_600 _of 0.62. We estimated that this culture contained 185 pM biotin based on comparison to the biotin standard curve depicted in Figure 6C. Thus, we obtained 37 pmol of biotin from the original 10 mL *E. coli *culture, and the specific activity of [^35^S]-biotin produced by our method is 30.7 Ci/mmol. In a previous attempt to label biotin with ^35^S in *Aspergillus niger*, Shimada and coworkers reported a specific activity of only 0.41 Ci/mmol[20]. We have thus achieved an increase in sensitivity of two orders of magnitude, which is a significant advantage for the detection of biotinylated molecules that are usually present at low intracellular concentrations. Although antibodies are available that can measure biotin, radiolabeled biotin is a more sensitive probe, and can be more accurately quantified. In addition, only radiolabeled biotin can track a specific population of biotin, and differentiate between newly synthesized biotin and biotin that is scavenged from the growth media.

## Conclusions

We developed a simple and effective strategy for the production of [^35^S]-biotin in *E. coli*. We have shown that a 79-residue fragment of the *P. falciparum *BCCP functions as a biotinylation substrate *in vivo*, and that the biotinylation efficiency of *Pf*BCCP-79 can be enhanced by overproduction of the *E. coli *biotin synthase, BioB, and biotin ligase, BirA. Biotinylated *Pf*BCCP-79 was purified from whole cell lysate using affinity chromatography, and free biotin was liberated by acid hydrolysis. We measured the reuptake of biotin in *E. coli *in order to quantify the biotin in the sample, and to verify that we had produced biologically active, radiolabeled [*D*]-biotin, which specifically labels cellular proteins.

## Methods

### Plasmid constructs for expression of *Pf*BCCP

The expression vector pGEXT was constructed from pGEX-4T-3 (GE Healthcare) by replacing the sequence encoding the thrombin cleavage site (beginning at base 918) with that of the tobacco etch virus (TEV) protease. This was done with the QuikChange site-directed mutagenesis kit (Stratagene), using primers TEV1, 5'-GGTGGCGACCATCCTCCAAAATCGGATgaaaacctgtattttcagggcGGATCCCCGAATTCCCGGGTC-3' and TEV2, 5'-GACCCGGGAATTCGGGGATCCgccctgaaaatacaggttttcATCCGATTTTGGAGGATGGTCGCCACC-3' (TEV cleavage site shown in lower case). The resulting vector, pGEXT, produces a glutathione *S*-transferase (GST) fusion protein which can be cleaved by TEV protease.

The biotin carboxy carrier protein (BCCP) domain (residues 1156-1369) of the *P. falciparum *acetyl-CoA carboxylase (ACC) gene [GenBank:XM_001348802; PlasmoDB:PF14_0664] was amplified by PCR from cDNA of *P. falciparum *3D7 erythrocyte stages, using primers BCCP1, 5'-GGTGGTGGATCCTTATGTGCTACCATATTTAAACTATTAATATATTTTATG-3' and BCCP2, 5'-GGTGGTCTCGAGTTATTCTATTATTCCTAATAAATCTCCTATTTTAATAATTG-3'. The PCR product was digested with *BamH*I and *Xho*I (underlined) and ligated into the pGEXT vector digested with the same endonucleases, generating plasmid pMSp038. The resulting fusion protein (*Pf*BCCP) contained the entire BCCP domain with an N-terminal GST tag. In order to test for solubility of the fusion protein, 100 ml cultures were grown to an OD_600 _of 0.6, protein expression was induced with IPTG, and the cultures were incubated for an additional three hours at 37°C. Cells were lysed, and whole cell lysate was centrifuged to separate the soluble and insoluble fractions. The *Pf*BCCP fusion protein was found in the insoluble fraction. A second construct was designed (*Pf*BCCP-79) based on the biotinylation domain found in the crystal structure of the *E. coli *BCCP [PDB:1BDO][38]. Nucleotides encoding ACC residues 1291-1369 were amplified from pMSp038 using primers BCCP3, 5'-GGTGGTGGATCCGATAATATTTTCATACCTAATGTTAGGAATCC-3' and BCCP2, digested with *BamH*I and *Xho*I and ligated into the pGEXT vector, generating plasmid pTDe003. Expression tests using pTDe003 indicated that the resulting GST fusion protein was soluble. *Pf*BCCP-79 was purified from *E. coli *(see below) and used for the generation of specific antisera in rabbits.

### Purification of *Pf*BCCP-79 for generation of rabbit antisera

Plasmid pTDe003, encoding GST-tagged *Pf*BCCP-79, was transformed into BL21-Star(DE3) cells (Invitrogen) previously transformed with the pRIL plasmid isolated from BL21-CodonPlus(DE3) cells (Stratagene). pRIL encodes rare tRNAs that aid in the expression of *P. falciparum *proteins in *E. coli*. Cells were grown to an OD_600 _of 0.8 in LB medium at 37°C, and then protein expression was induced with 0.4 mM IPTG and the cultures were maintained at 20°C for 10 hrs. Cells were harvested by centrifugation at 4,000 g for 20 min at 4°C followed by resuspension in 20 mL of lysis buffer (phosphate buffered saline (PBS) solution pH 7.5, 1 mg/mL lysozyme, 2.5 μg/mL DNAse I, 10 mM PMSF, 10 mM DTT) per liter of cell culture. The resuspended cell mixture was sonicated, and then cleared by centrifugation at 30,000 g for 20 min at 4°C. Cleared supernatant was loaded on a GSTrap™ Fast-Flow chromatography column (GE Healthcare) equilibrated in PBS pH 7.5. After washing with 5 column volumes of PBS, GST fusion protein was eluted with 5 mM reduced glutathione in equilibration buffer. Fractions containing fusion protein were pooled and digested with 10 μg/ml TEV protease for 6 days at 4°C. The protein sample was dialyzed to remove glutathione and then re-applied to the GSTrap™ FF column to remove the liberated GST. The purified protein was concentrated to 1.185 g/L with a 5000 MW cutoff concentrator (Vivascience) and sent to Cocalico Biologicals, Inc. for production of specific antisera in rabbits.

Polyclonal antibodies were raised against *Pf*BCCP-79 according to the standard immunization protocol specified by Cocalico Biologicals, Inc. Prebleeds from four rabbits were screened for cross-reactivity to *P. falciparum *and human red blood cell antigens by western blot analysis. The two rabbits that were least reactive were selected for inoculation with 100 μg purified *Pf*BCCP-79. The rabbits were boosted with 50 μg antigen on days 14, 21 and 49 after initial inoculation. Test bleeds were performed on days 35 and 56, and were tested for reactivity to the *Pf*BCCP-79 antigen by western blot analysis. Production bleeds were performed on days 63 and 84, and the rabbits were exsanguinated on day 91. These antisera were used without further purification for western blot analysis, as described below.

### Plasmid constructs for expression of biotinylated *Pf*BCCP-79

For labeling with ^35^S, the GST fusion protein is undesirable due to the presence of multiple cysteine residues that will incorporate the label. To address this problem, we constructed a plasmid expressing *Pf*BCCP-79 with a six-histidine tag. The *Pf*BCCP-79 gene sequence was amplified from pTDe003 using primers BCCP4, 5'-GGTGGTCATATGGATAATATTTTCATACCTAATGTTAGGAATCC-3' and BCCP5, 5'-GGTGGTGAATTCTTATTCTATTATTCCTAATAAATCTCCTATTTTAATAATTG-3', digested with *Nde*I and *Eco*RI (underlined) and ligated into the pET28a vector (Invitrogen), generating plasmid pTDe010. This plasmid contains a kanamycin resistance cassette and a pMB1 origin of replication, and produces His-tagged *Pf*BCCP-79 under control of a T7 promoter.

Plasmid pDB1282 was a gift from Dennis Dean at Virginia Polytechnic Institute and State University (Virginia Tech). Plasmid pDB1282 contains the Isc (Iron-Sulfur Cluster) operon from *Azotobacter vinelandii*, which is required for iron-sulfur cluster biogenesis in this organism[30]. The *Azotobacter vinelandii *Isc operon is composed of the genes *iscR, iscS, iscU, iscA, hscB, hscA, fdx *and *iscX*[39]. The essential genes of this cluster (*iscS, iscU, iscA, hscB, hscA *and *fdx*)[39] were cloned into a variant of the pARA13 expression vector[40] to form pDB1282. Sequencing of the 5' and 3' splice sites suggests that the Isc operon was restricted with *Bsp*HI and ligated into the unique *Nco*I site of pAra13. Interestingly, pDB1282 contains the seven complete genes from *iscS *to *iscX *as well as about 200 nucleotides of a downstream nucleoside-diphosphate kinase (*ndk*). This plasmid contains an ampicillin resistance cassette and produces the Isc genes under control of an arabinose-inducible promoter.

Plasmid pCY216 [GenBank:AAD22470.1][23] was a gift from John Cronan at the University of Illinois at Urbana-Champaign. It was modified from the pARA13 expression vector, and encodes *E. coli *BirA under control of an arabinose-inducible promoter in a chloramphenicol resistance plasmid containing a p15a origin of replication.

Plasmid pRK586[41] was modified to produce the *E. coli *biotin synthase (BioB). The *bioB *gene was amplified from K-12 *E. coli *with the primers BioB1, 5'-GGTGGTGGTACCATGCATATGGCTCACCGCCCACGCTG-3', and BioB2, 5'-GGTGGTGGATCCGCGGCCGCTCATAATGCTGCCGCGTTGTAATATTC-3'. The resulting amplicon, which contained 5' *Kpn*I/*Nsi*I sites and 3' *Not*I/*Bam*HI sites (underlined), was digested with *Kpn*I and *Bam*HI and ligated into pRK586 digested with the same endonucleases. The resulting plasmid, pSPr058, was digested with *Bcl*I and *Sac*I to excise the kanamycin resistance gene (encoding aminoglycoside 3'-phosphotransferase). Primers Amp1, 5'-GGTGGTTGATCAGCCTTTTTGCGTTTCTACAAACTC-3' and Amp2, 5'-GGTGGTGAGCTCTTACCAATGCTTAATCAGTGAGGC-3' were used to amplify the ampicillin resistance gene (encoding β-lactamase) and 118 upstream bases from plasmid pDB1282. The resulting amplicon was digested with *Bcl*I and *Sac*I (underlined) and ligated into the pSPr058 fragment described above. The resulting plasmid, pSPr059, encodes BioB under control of the λP_L/tetO _promoter in an ampicillin resistant plasmid containing a pSC101 origin of replication.

### *In vivo *biotinylation of *Pf*BCCP-79 and western blotting

The methionine auxotroph *E. coli *strain B834(DE3) (Novagen) was transformed with plasmid pTDe010 (encoding *Pf*BCCP-79), in combination with either pSPr059 (encoding BioB), pCY216 (encoding BirA) or pDB1282 (encoding the *Av*Isc proteins). Transformed cells were grown to an OD_600 _of 0.8 at 37°C with the appropriate antibiotics in minimal medium composed of sulfur-free minimal E medium (0.83 mM MgCl_2_, 9.5 mM citric acid, 58 mM K_2_HPO_4_, 29.7 mM NH_4_Cl, 16.7 mM NaH_2_PO_4_) supplemented with 0.4% (w/v) glucose, 100 μM FeCl_3_, and 2 mM methionine[42]. Protein expression was induced with 0.4 mM IPTG and 0.025% arabinose, and the BioB substrate desthiobiotin (Sigma-Aldrich) was added at a concentration of 1 mM. Cells were grown for 10 hours at 20°C. Cultures were normalized based on their cell density, and equal amounts of cells were harvested by centrifugation at 16,000 g for 5 min in 1.5 ml Eppendorf tubes. Cell pellets were lysed in NuPAGE sample buffer (Invitrogen) and vortexed to shear genomic DNA. For western blot analysis, proteins were separated by sodium dodecyl sulfate polyacrylamide gel electrophoresis (SDS-PAGE) on 4-12% bis-tris acrylamide gradient gels. The gels were blotted onto 0.2 μm nitrocellulose membranes (Invitrogen) for 120 min at 5 V using a Semi-Dry transfer cell (BioRad). Membranes were blocked in 5% non-fat dry milk (Carnation) in PBS, washed in PBS, and probed with 1:4,000 streptavidin-HRP, ultrasensitive (Sigma-Aldrich). After PBS washes, HRP was detected using the Supersignal^® ^West Pico chemiluminescent kit (Pierce). Membranes were then stripped using 4% (w/v) trichloroacetic acid (TCA), blocked as before, washed in PBS, and probed with rabbit antiserum specific for *Pf*BCCP-79 (1:4,000). After PBS washes, the blot was probed with 1:5,000 donkey anti-rabbit immunoglobulin antibody conjugated to horseradish peroxidase (GE Healthcare). Excess antibody was removed with PBS, and HRP was detected using the chemiluminescent kit.

### Expression and purification of [^35^S]-biotin-*Pf*BCCP-79

For radiolabeling studies, the methionine auxotroph *E. coli *strain B834(DE3) (Novagen) was transformed with plasmids pTDe010, pSPr059 and pCY216. Cells were cultured at 37°C in 10 mL sulfur-free minimal medium (described above). When the culture reached an OD_600 _of 0.8, expression of *Pf*BCCP-79 and BirA were induced with 0.4 mM IPTG and 0.025% arabinose, respectively. The culture was transferred to 20°C, and after one hour, 1 mCi Na^35^SO_4 _(American Radiolabeled Chemicals, 10 mCi/ml) and 1 mM desthiobiotin were added. The culture was maintained at 20°C for an additional 10 hours. Cells were then harvested by centrifugation for 20 min at 3,000 g and frozen at -20°C for later purification.

Cell pellets were lysed at room temperature for 10 min in 1 mL BugBuster (Novagen) supplemented with 1 mg/mL lysozyme and 2.5 μg/mL DNAseI. The cell lysate was cleared by centrifugation for 5 min at 16,000 g and applied to a 1 ml HiTrap Metal Chelate HP Column (GE Healthcare) equilibrated with Buffer A (20 mM Na/K phosphate pH 7.5). The column was washed with 7 mL Buffer A, and the combined flow through was collected and labeled FT1. The column was washed with 5 mL 30 mM imidazole in Buffer A (W1), followed by 5 mL 1 M NaCl in Buffer A (W2), and 5 mL 1% Triton X-100 in Buffer A (W3). The metal chelate column was then connected to a 4.6 mm × 100 mm PEEK™column (Applied Biosystems) packed with 1.7 mL SoftLink™ Soft Release Avidin Resin (Promega), and bound proteins were eluted from the metal chelate column with 5 mL 400 mM imidazole in Buffer A (FT2). Both columns in tandem were washed with 8 mL Buffer A (W4), and then the metal chelate column was removed. Bound protein was eluted from the avidin column in 4 mL 50 mM glycine pH 2.9 (E1). 400 μl 1 M Tris was added to the collection tube prior to eluting to prevent damage over time from the low pH of the elution buffer.

The purified biotinylated *Pf*BCCP-79 protein was precipitated by adding 100% (w/v) TCA to a final concentration of 10%, and centrifuging for 15 min at 16,000 g. The resulting protein pellet was resuspended in 200 μL 5 M HCl and incubated at 95°C for 8 hours. One μL 1 g/L phenol red was added as a pH indicator, and the resulting hydrolysate was buffered by adding 50 μL 1 M K_2_HPO_4_, and neutralized by adding 10 M NaOH until the pH indicator turned pink.

### Verification and quantification of biologically active [^35^S]-biotin in *E. coli*

A biotin bioassay was developed using a biotin auxotroph *E. coli *strain in which the biotin synthase (*bioB*) gene had been replaced with a kanamycin resistance cassette (National BioResource Project (NIG, Japan):Keio JW0758). These cells were grown to full density in LB with 50 μg/mL kanamycin. To deplete biotin levels prior to the assay, 1 μL of the LB culture was added to 4 mL biotin-free minimal medium composed of minimal E medium (0.81 mM MgSO_4_, 9.5 mM citric acid, 58 mM K_2_HPO_4_, 7.4 mM (NH_4_)_2_SO_4_, 16.7 mM NaH_2_PO_4_) supplemented with 0.4% (w/v) glucose, 30 μM FeSO_4_, and 50 μg/mL kanamycin. This culture grew to full density over a one day period at 37°C, and 1 μL of this culture was used to start a second 4 mL culture in biotin-free minimal medium. This culture achieved a lower density, presumably due to the 16,000,000 fold dilution of biotin from the original LB culture. For the bioassay, 2 μL of the biotin depleted culture was added to 2 mL biotin-free minimal medium supplemented with known concentrations of biotin. Assay cultures were grown at 37°C for 16 hours, after which the optical density was measured at 600 nm. These cultures were used to construct a standard growth curve. Parallel cultures were supplemented with serial dilutions of *Pf*BCCP-79 hydrolysate. Cell densities of cultures supplemented with the hydrolysate were compared with the standard curve in order to quantify the biologically active biotin in the hydrolysate. In order to detect [^35^S]-biotin incorporation into the *E. coli *BCCP protein, cells were harvested and proteins were separated by SDS-PAGE, transferred to nitrocellulose membrane and exposed to autoradiography film.

## Authors' contributions

TD performed the experiments, analyzed the data, and helped to draft the manuscript. MS cloned the *Pf*BCCP domain from cDNA, performed the solubility test, and helped generate the anti-BCCP antibody. SP conceived of the study, and participated in its design and coordination and helped to draft the manuscript. All authors read and approved the final manuscript.
